# Causal association of circulating cytokines with sepsis: a Mendelian randomization study

**DOI:** 10.3389/fimmu.2023.1281845

**Published:** 2023-10-17

**Authors:** Shan Lin, Xueyan Mao, Wanmei He

**Affiliations:** ^1^ Department of Respiratory and Critical Care Medicine, Affiliated Hospital of North Sichuan Medical College, Nanchong, Sichuan, China; ^2^ Department of Medical Intensive Care Unit, The First Affiliated Hospital of Sun Yat-Sen University, Guangzhou, Guangdong, China

**Keywords:** Mendelian randomization, cytokines, sepsis, causality, genome-wide association study

## Abstract

**Background:**

Observational studies have reported an association between circulating cytokines and sepsis. However, the precise causal relationship between these factors remains unclear. The objective of this study was to explore the causal link between circulating cytokines and sepsis using genetic data within the framework of Mendelian Randomization (MR).

**Methods:**

We performed a two-sample MR analysis to investigate this causality relationship in individuals of European ancestry. The publicly available genome-wide association studies (GWAS) statistics were used. We selected eligible instrumental single nucleotide polymorphisms (SNPs) that were significantly related to the circulating cytokines. Multiple MR analysis approaches were carried out, which included inverse variance weighted (IVW), Weighted Median, MR-Egger, Weighted Mode, Simple Mode, and MR pleiotropy residual sum and outlier (MR-PRESSO) methods.

**Results:**

We found evidence to support the causal role of genetically predicted circulating levels on decreased risk of sepsis, including RANTES (OR = 0.920, 95% CI: 0.849-0.997, *P* = 0.041) and basic fibroblast growth factor (basic-FGF) (OR = 0.869, 95% CI: 0.766-0.986, *P* = 0.029). Additionally, MR analysis positive causal association of between beta-nerve growth factor (β-NGF) and sepsis (OR = 1.120, 95% CI: 1.037-1.211, *P* = 0.004). The results of MR-Egger, Weighted Median, Weighted Mode, and Simple Mode methods were consistent with the IVW estimates. Sensitivity analysis showed no horizontal pleiotropy to bias the causal estimates.

**Conclusion:**

This MR study provides first novel evidence that genetically predicted causal association of circulating levels of RANTES, basic-FGF, and β-NGF with altered sepsis risk. The findings shed light on the potential involvement of these cytokines in sepsis pathogenesis. Although requiring additional confirmation, the results contribute new insights into cytokine mediators in sepsis and suggest promising future research directions.

## Introduction

Sepsis, a systemic inflammatory response syndrome triggered by the body in response to pathogens, is a critical condition that demands prompt and effective treatment. Failure to intervene in a timely manner can result in the dysfunction of multiple organs and a mortality rate ranging from 30% to 50% (1). Epidemiological studies have shed light on the global prevalence of sepsis, with approximately 17 million cases reported in 2017, showing a notable decrease of 18.8% compared to the 60.2 million cases reported in 1990 (2). The alarming impact of sepsis is further highlighted by estimations of 11 million sepsis-related deaths worldwide in 2017, making up a staggering 19.7% of all deaths that year (2). These statistics underscore the urgent need for heightened awareness, improved prevention strategies, and enhanced treatment options to combat this life-threatening condition.

Inflammation lies at the heart of sepsis pathophysiology (3). While an inflammatory response is vital to combat infection initially, excessive inflammation can inflict damage on the host (4). This explains the early use of glucocorticoids in sepsis to temper harmful hyperinflammation (5, 6). However, as sepsis progresses, the inflammatory response wanes while immunosuppression comes to the fore (7). Prolonged glucocorticoid administration then risks precipitating immune paralysis (8). The inflammatory cascade in sepsis is driven by the release of potent cytokines including TNF-α, interleukin (IL)-1, and IL-6, as well as other mediators like chemokines, complement, and reactive oxygen species (4, 9–12). The ensuing dysregulated immune response and tissue injury arise from complex interplay between inflammatory pathways (13). Though research continues to unravel these nuanced relationships, the precise causal mechanisms remain enigmatic. Recent advances harnessing models and technology have offered fresh insights, while clinical studies illuminated potential anti-inflammatory therapeutic targets (14, 15). In summary, despite copious evidence linking inflammatory mediators to sepsis pathogenesis, the intricate inflammatory imbalance underpinning sepsis progression remains incompletely understood.

Mendelian randomization (MR) is an epidemiological technique that utilizes genetic variants as instrumental variables (IVs) to assess causal relationships between modifiable exposures and disease outcomes (16). The random assortment of alleles at conception results in a random distribution of genotypes in the population. Therefore, genetic variants can be leveraged as unconfounded proxies for exposures of interest. By examining the association between genetic variants linked to exposures and disease risk, MR can infer causality while minimizing biases from reverse causation and confounding that afflict conventional observational studies (17).

In this study, we aimed to employ MR to explore the causal nature of the relationship between circulating cytokines and sepsis. By using genetic instruments as proxies for circulating cytokines, we can assess whether inflammation has a causal role in sepsis development or merely represents an epiphenomenon. Elucidating these causal pathways will provide greater biological insight and may uncover novel therapeutic targets for this deadly syndrome.

## Method

### Study design

**Figure 1:**
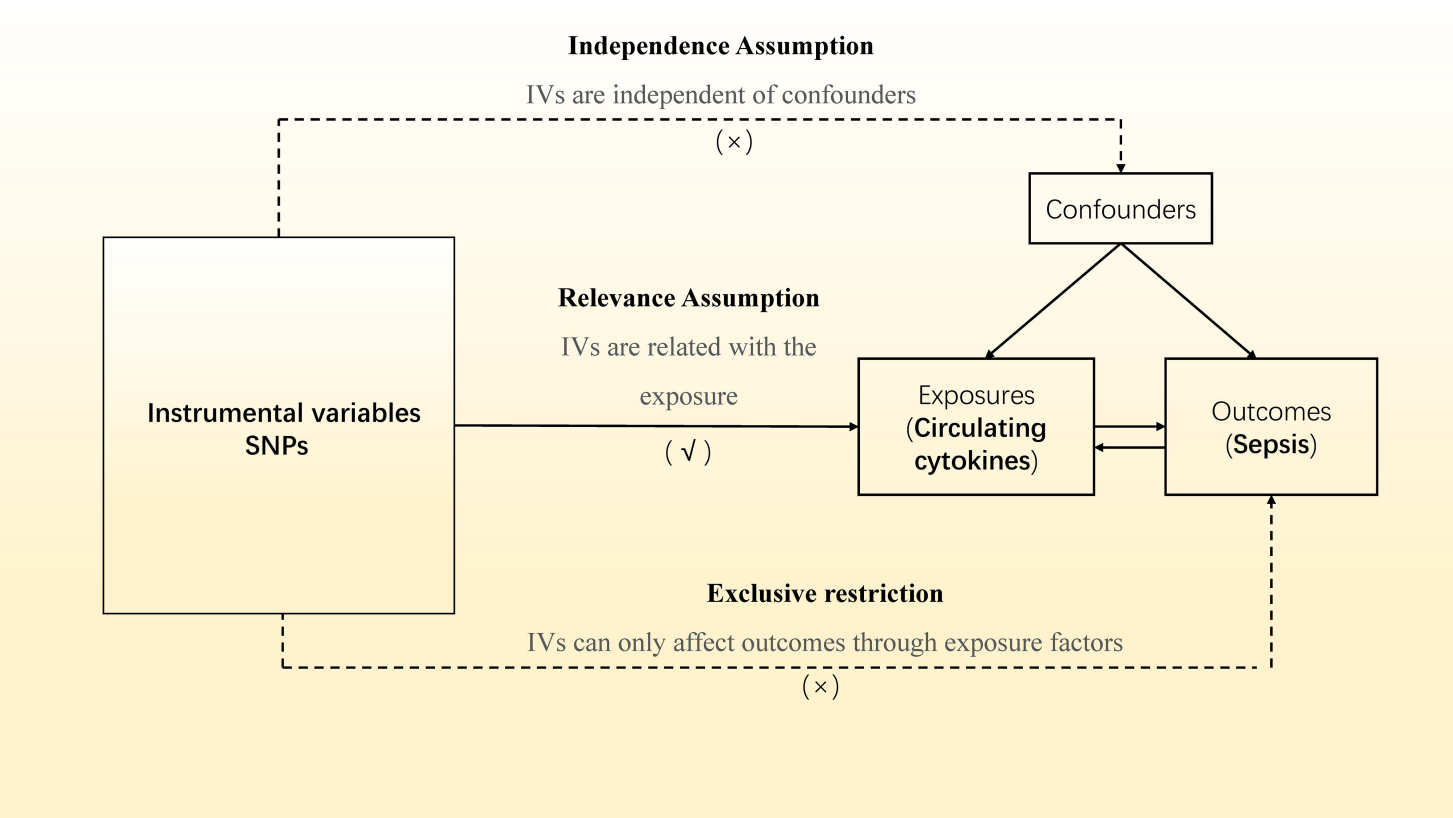
Overview of the current Mendelian randomization (MR) study. SNPs, single nucleotide polymorphisms; IVs, instrumental variables. A √ symbol indicates that the criteria was achieved; a × symbol indicates that the criteria was not achieved.

In our two-sample MR study (**Figure 1**), single nucleotide polymorphisms (SNPs) were exploited as instrument variables (IVs). To ensure the data validity, we chose SNPs through three major assumptions: (1) IVs should be significantly associated with the exposure factors (‘‘Relevance assumption’’); (2) IVs affect the outcomes only via exposure factors rather than the other pathways, which implies no horizontal pleiotropy (‘‘Exclusivity assumption’’); (3) IVs were not relevant to any confounding factors (‘‘Independence assumption’’) (18).

### Data resource

SNPs on circulating cytokines and sepsis was selected from the latest genome-wide association study (GWAS), as presented in **Supplementary Table 1**.

**Figure 1** depicts the study’s overview in detail. Summary data from the most thorough and extensive cytokine GWAS were used for the genetic tool of cytokines. The cytokine GWAS meta-analysis included 8,293 Finns from three distinct population-based cohorts: the Young Finns Cardiovascular Risk Study, FINRISK1997, and FINRISK2002 studies (19). Participants were chosen at random from five distinct geographic regions and between the ages of 25 and 74 during the survey’s administration in Finland. The subjects’ EDTA plasma, heparin plasma, and blood were tested for cytokine levels. Only observations within each cytokine’s detectable range and cytokines with more than 90% of their values missing were removed from the study (7 out of 48). Written informed permission was given by each subject.

To explore the causal effect of exposure of various circulating cytokines on the risk of sepsis, we selected datasets for sepsis as the outcome from the summary statistics of the GWAS from UK Biobank (n = 11,643 for sepsis case, n = 474,841 for control). Notably, sepsis cases were identified by International Classification of Diseases (ICD), 10th edition, codes A02, A39, A40, and A41, aligning with definitions used in recent research (20–22). Cases were included when these codes appeared in either the primary or secondary diagnostic position within hospital episode statistics data, or similar datasets from devolved nations, as provided by UK Biobank. Then, using GWAS summary statistics, we used two-sample MR methods to deduce the causative link between inflammatory factors and sepsis. Since samples of inflammatory regulators and sepsis were acquired from various consortiums, there was no overlap.

### SNPs selection

We performed a set of methods to filter valid SNPs that suit the three core MR assumptions. Firstly, the independent SNPs strongly linked to different circulating cytokines were selected (23, 24). SNPs with P-value < 5×10^-6^ were considered to be significantly associated with circulating cytokines to obtain more SNPs as IVs. Secondly, we adopted the clumping process to evaluate the linkage disequilibrium (LD) among the SNPs (r^2^< 0.001 and clumping distance = 10,000 kb). The SNPs with LD were removed to avoid biased results. Thirdly, we searched all the screened SNPs on PhenoScanner V2 (http://www.phenoscanner.medschl.cam.ac.uk/) (25). PhenoScanner V2 provides the phenotypes information of SNPs, which can be used to determine whether the SNPs only affect the outcomes through exposure. The SNPs related to the confounding factors, such as smoking, diabetes and worries, were excluded to eliminate the bias. Finally, we harmonized the exposure and outcome datasets to remove the non-concordant SNPs. The remaining SNPs were used as the genetic IVs.

Moreover, the F statistics for the SNPs were calculated by the following equation: *F* = *R* ^2^ × (*N*–2)/(1–*R* ^2^). R^2^ was the proportion of variance. N represented the sample size. Weak instruments were identified by IVs with an F statistic less than 10 (F < 10) and excluded from the analysis (26).

### Statistical analysis

After selecting the valid SNPs, we adopted inverse variance weighted (IVW) as the main way to estimate the MR analysis. IVW assesses the overall causal impact of exposure on the outcomes. It is the most accurate way to evaluate causality if all the selected SNPs are valid (27). We also applied complementary methods to analyze causal association, including Weighted Median, MR Egger, Weighted Mode, and Simple Mode methods. The Weighted Median method will generate a more potent effect when more than half of the SNPs are valid (28). MR Egger provides accurate effect estimates even if all the SNPs are invalid (29).

We further conducted the MR-Egger regression and the MR Pleiotropy Residual Sum and Outlier (MR-PRESSO) test to evaluate the possible horizontal pleiotropy (30, 31). In the MR-Egger regression, the intercept term indicates the average pleiotropic effect of IVs (31). We used Cochran’s Q statistic and MR-egger regression to test the heterogeneities. Additionally, the leave-one-out analysis was utilized to assess the robustness and consistency of the results.

All the analyses were performed with the packages “Two Sample MR” and “MRPRESSO” in R version 4.2.1. P < 0.05 is statistically significant.

## Results

### Causal effect of circulating cytokines on sepsis

After the series of filters mentioned in the method, 4-16 SNPs were left as IVs for circulating cytokines (**Supplementary Table 1**). All the selected SNPs were robust instruments, as confirmed by the F-statistic values being more than 10.

**Figure 2:**
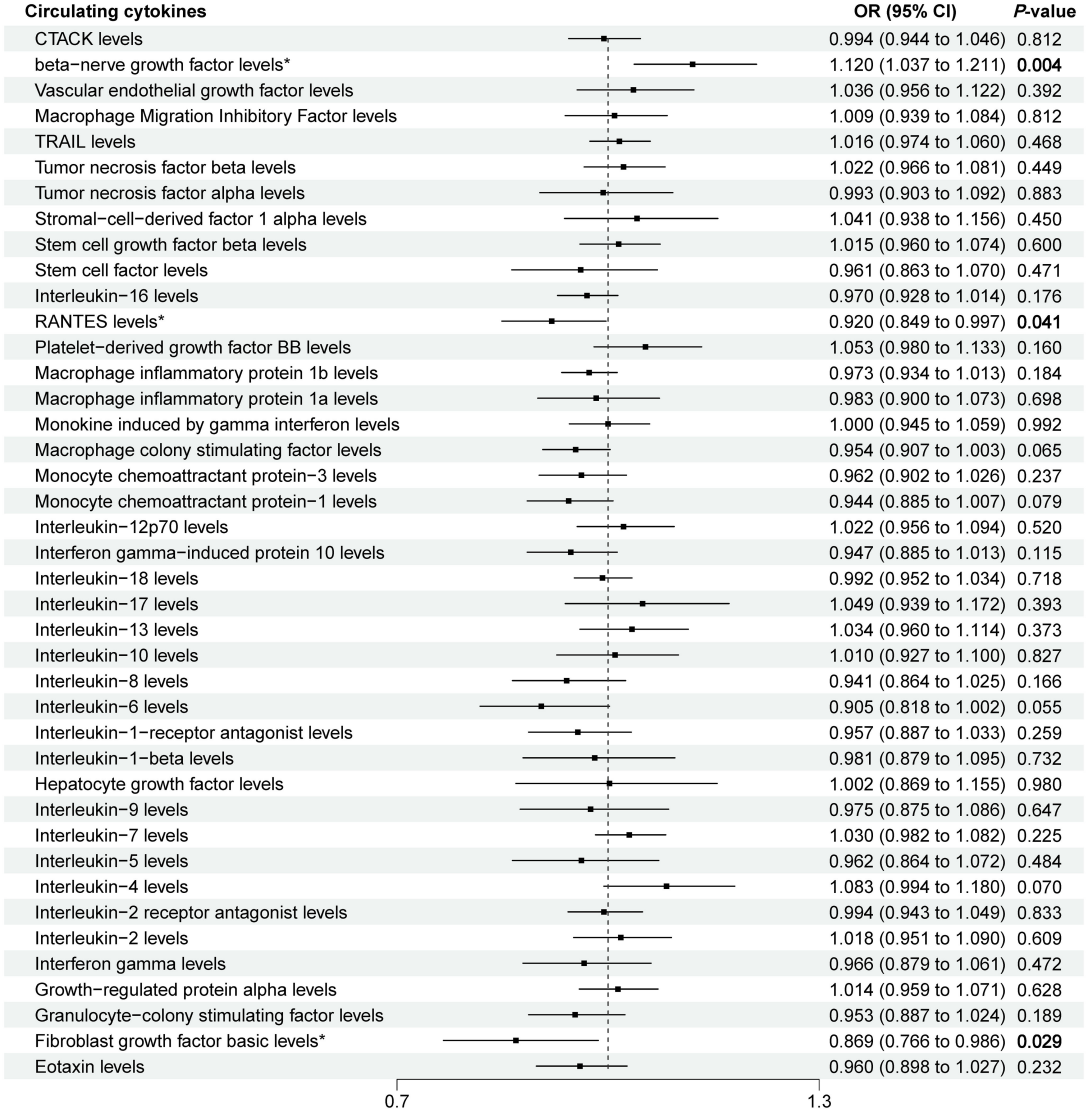
Forest plot for the causal effect of circulating cytokines on the risk of sepsis derived from IVW. OR, odds ratio; CI, confdence interval; IVW, inverse variance weighting; CTACK, cutaneous T-cell attracting (CCL27); RANTES, regulated on activation, normal T-cell expressed and secreted (CCL5); TRAIL TNF-related apoptosis-inducing ligand. The * symbol represents a p-value of less than 0.05, which is statistically significant. Bold values represent p-values less than 0.05, which is statistically significant.

Next, we adopted these SNPs to analyze the causal link. The MR estimates between circulating cytokines and sepsis of different methods are presented in (**Supplementary Table 2**). Specifically, the preliminary results of IVW revealed negative causal effect of two cytokines on sepsis, including RANTES (regulated on activation, normal T-cell expressed and secreted (CCL5)) [OR = 0.920, 95% CI: 0.849-0.997, *P* = 0.041] and basic fibroblast growth factor (basic-FGF) [OR = 0.869, 95% CI: 0.766-0.986, *P* = 0.029], and positive causal effect of beta-nerve growth factor (β-NGF) and sepsis [OR = 1.120, 95% CI: 1.037-1.211, *P* = 0.004] (**Table 1** and **Figure 2**). In addition, the MR-Egger, Weighted Median method showed consistent results. The scatter plots demonstrated the specific effects of each method per outcome database (**Supplementary Figures 1**, **3**, **5**).

**Table 1:** Primary results for MR analysis of circulating cytokines on sepsis.

	Method	Beta	SE	OR	95% CI	*P* -value
CTACK levels	Inverse variance weighted	-0.006	0.026	0.994	0.944-1.046	0.812
beta-nerve growth factor levels*	Inverse variance weighted	0.114	0.040	1.120	1.037-1.211	**0.004**
Vascular endothelial growth factor levels	Inverse variance weighted	0.035	0.041	1.036	0.956-1.122	0.392
Macrophage Migration Inhibitory Factor levels	Inverse variance weighted	0.009	0.037	1.009	0.939-1.084	0.812
TRAIL levels	Inverse variance weighted	0.016	0.022	1.016	0.974-1.060	0.468
Tumor necrosis factor beta levels	Inverse variance weighted	0.022	0.029	1.022	0.966-1.081	0.449
Tumor necrosis factor alpha levels	Inverse variance weighted	-0.007	0.049	0.993	0.903-1.092	0.883
Stromal-cell-derived factor 1 alpha levels	Inverse variance weighted	0.040	0.053	1.041	0.938-1.156	0.450
Stem cell growth factor beta levels	Inverse variance weighted	0.015	0.029	1.015	0.960-1.074	0.600
Stem cell factor levels	Inverse variance weighted	-0.040	0.055	0.961	0.863-1.070	0.471
Interleukin-16 levels	Inverse variance weighted	-0.031	0.023	0.970	0.928-1.014	0.176
RANTES levels*	Inverse variance weighted	-0.083	0.041	0.920	0.849-0.997	**0.041**
Platelet-derived growth factor BB levels	Inverse variance weighted	0.052	0.037	1.053	0.980-1.133	0.160
Macrophage inflammatory protein 1b levels	Inverse variance weighted	-0.027	0.021	0.973	0.934-1.013	0.184
Macrophage inflammatory protein 1a levels	Inverse variance weighted	-0.017	0.045	0.983	0.900-1.073	0.698
Monokine induced by gamma interferon levels	Inverse variance weighted	0.000	0.029	1.000	0.945-1.059	0.992
Macrophage colony stimulating factor levels	Inverse variance weighted	-0.047	0.026	0.954	0.907-1.003	0.065
Monocyte chemoattractant protein-3 levels	Inverse variance weighted	-0.039	0.033	0.962	0.902-1.026	0.237
Monocyte chemoattractant protein-1 levels	Inverse variance weighted	-0.058	0.033	0.944	0.885-1.007	0.079
Interleukin-12p70 levels	Inverse variance weighted	0.022	0.035	1.022	0.956-1.094	0.520
Interferon gamma-induced protein 10 levels	Inverse variance weighted	-0.055	0.035	0.947	0.885-1.013	0.115
Interleukin-18 levels	Inverse variance weighted	-0.008	0.021	0.992	0.952-1.034	0.718
Interleukin-17 levels	Inverse variance weighted	0.048	0.056	1.049	0.939-1.172	0.393
Interleukin-13 levels	Inverse variance weighted	0.034	0.038	1.034	0.960-1.114	0.373
Interleukin-10 levels	Inverse variance weighted	0.010	0.044	1.010	0.927-1.100	0.827
Interleukin-8 levels	Inverse variance weighted	-0.060	0.044	0.941	0.864-1.025	0.166
Interleukin-6 levels	Inverse variance weighted	-0.100	0.052	0.905	0.818-1.002	0.055
Interleukin-1-receptor antagonist levels	Inverse variance weighted	-0.044	0.039	0.957	0.887-1.033	0.259
Interleukin-1-beta levels	Inverse variance weighted	-0.019	0.056	0.981	0.879-1.095	0.732
Hepatocyte growth factor levels	Inverse variance weighted	0.002	0.073	1.002	0.869-1.155	0.980
Interleukin-9 levels	Inverse variance weighted	-0.025	0.055	0.975	0.875-1.086	0.647
Interleukin-7 levels	Inverse variance weighted	0.030	0.025	1.030	0.982-1.082	0.225
Interleukin-5 levels	Inverse variance weighted	-0.039	0.055	0.962	0.864-1.072	0.484
Interleukin-4 levels	Inverse variance weighted	0.080	0.044	1.083	0.994-1.180	0.070
Interleukin-2 receptor antagonist levels	Inverse variance weighted	-0.006	0.027	0.994	0.943-1.049	0.833
Interleukin-2 levels	Inverse variance weighted	0.018	0.035	1.018	0.951-1.090	0.609
Interferon gamma levels	Inverse variance weighted	-0.035	0.048	0.966	0.879-1.061	0.472
Growth-regulated protein alpha levels	Inverse variance weighted	0.014	0.028	1.014	0.959-1.071	0.628
Granulocyte-colony stimulating factor levels	Inverse variance weighted	-0.048	0.037	0.953	0.887-1.024	0.189
Fibroblast growth factor basic levels*	Inverse variance weighted	-0.141	0.065	0.869	0.766-0.986	**0.029**
Eotaxin levels	Inverse variance weighted	-0.041	0.034	0.960	0.898-1.027	0.232

MR, mendelian randomization; SE, standard error; OR, odds ratio; CI, confdence interval; CTACK, cutaneous T-cell attracting (CCL27); RANTES, regulated on activation, normal T-cell expressed and secreted (CCL5); TRAIL TNF-related apoptosis-inducing ligand.

The * symbol represents a p-value of less than 0.05, which is statistically significant.

Bold values represent p-values less than 0.05, which is statistically significant.

The MR-Egger regression and MR-PRESSO global test results revealed no horizontal pleiotropy (**Table 2**; **Supplementary Table 3**). More importantly, Cochran’s Q statistic and MR-egger regression showed no heterogeneity between the individual SNPs (*P* > 0.05). Additionally, the p-values of the MR PRESSO global test for circulating cytokines on sepsis were all greater than 0.05. The leave-one-out analysis further confirmed that the causal estimates of circulating cytokines were given (**Supplementary Figures 2**, **4**, **6**).

Apart from RANTES, basic-FGF and β-NGF, the other 38 cytokines (e.g., GRO-α, Trail, MIG, IL-17) did not show any association with the risk of sepsis in either IVW primary MR analysis or in other secondary analyses (**Supplementary Table 2**). In the heterogeneity assay, most of the cytokines were significantly non-heterogeneous, except for hepatocyte growth factor (*P* = 0.022). MR-egger regression did not show pleiotropy in p values for all cytokines (**Table 2**). An additional solidity test, the MR-PRESSO assay, did not show any abnormal values for the significant MR results (**Supplementary Table 3).**

**Table 2:** Heterogenity and pleiotropy analyses of circulating cytokines on sepsis.

	Heterogenity		MR-Egger intercept		
	Q	Q_*P* -value	Egger_intercept	SE	*P* -value
CTACK levels	10.741	0.465	0.007	0.014	0.638
beta-nerve growth factor levels	4.776	0.687	-0.014	0.032	0.681
Vascular endothelial growth factor levels	7.061	0.631	-0.021	0.020	0.319
Macrophage Migration Inhibitory Factor levels	4.582	0.801	0.017	0.016	0.342
TRAIL levels	18.536	0.293	-0.004	0.009	0.691
Tumor necrosis factor beta levels	4.239	0.237	0.018	0.014	0.322
Tumor necrosis factor alpha levels	5.675	0.225	-0.027	0.015	0.178
Stromal-cell-derived factor 1 alpha levels	9.083	0.335	0.002	0.012	0.851
Stem cell growth factor beta levels	18.002	0.207	-0.003	0.011	0.766
Stem cell factor levels	15.325	0.121	-0.013	0.016	0.432
Interleukin-16 levels	6.444	0.598	0.013	0.015	0.410
RANTES levels	4.965	0.664	0.012	0.021	0.584
Platelet-derived growth factor BB levels	17.646	0.171	-0.013	0.011	0.268
Macrophage inflammatory protein 1b levels	12.883	0.845	0.004	0.008	0.649
Macrophage inflammatory protein 1a levels	2.804	0.833	0.020	0.021	0.391
Monokine induced by gamma interferon levels	15.453	0.280	0.010	0.015	0.516
Macrophage colony stimulating factor levels	4.539	0.806	-0.009	0.017	0.612
Monocyte chemoattractant protein-3 levels	0.914	0.822	-0.003	0.029	0.932
Monocyte chemoattractant protein-1 levels	11.304	0.662	-0.013	0.010	0.232
Interleukin-12p70 levels	19.607	0.105	-0.008	0.010	0.401
Interferon gamma-induced protein 10 levels	6.051	0.642	-0.012	0.013	0.394
Interleukin-18 levels	11.363	0.878	0.002	0.009	0.860
Interleukin-17 levels	18.474	0.071	0.030	0.018	0.125
Interleukin-13 levels	8.215	0.314	-0.014	0.017	0.434
Interleukin-10 levels	15.142	0.127	-0.014	0.012	0.252
Interleukin-8 levels	1.225	0.747	0.001	0.014	0.960
Interleukin-6 levels	6.024	0.537	-0.015	0.014	0.323
Interleukin-1-receptor antagonist levels	6.355	0.608	0.017	0.017	0.338
Interleukin-1-beta levels	1.192	0.879	-0.020	0.019	0.371
Hepatocyte growth factor levels	16.328	0.022	-0.029	0.028	0.338
Interleukin-9 levels	11.663	0.070	0.019	0.029	0.552
Interleukin-7 levels	6.919	0.806	-0.010	0.016	0.538
Interleukin-5 levels	4.865	0.301	-0.017	0.025	0.540
Interleukin-4 levels	8.296	0.600	0.012	0.011	0.311
Interleukin-2 receptor antagonist levels	1.875	0.966	-0.001	0.012	0.951
Interleukin-2 levels	9.779	0.369	0.007	0.012	0.557
Interferon gamma levels	13.637	0.325	0.014	0.012	0.275
Growth-regulated protein alpha levels	13.213	0.105	0.010	0.022	0.672
Granulocyte-colony stimulating factor levels	8.435	0.392	-0.017	0.011	0.162
Fibroblast growth factor basic levels	4.850	0.678	0.010	0.017	0.592
Eotaxin levels	10.516	0.724	0.005	0.012	0.661

SE, standard error; CTACK, cutaneous T-cell attracting (CCL27); RANTES, regulated on activation, normal T-cell expressed and secreted (CCL5); TRAIL TNF-related apoptosis-inducing ligand.

### No causal effect of sepsis on circulating cytokines

To further explore the causal effect of sepsis on the circulating cytokines, we selected independent SNPs as IVs for sepsis. The F-statistic values were all higher than 10, which confirmed that all the selected SNPs were valid instruments. The results of the IVW analysis confirmed no causal effect of sepsis on all circulating cytokines (**Table 3**). Furthermore, most results revealed no heterogeneities based on the results of Cochran’s Q statistic, except for macrophage inflammatory protein 1b (*P* = 0.009). Horizontal pleiotropy was not detected in the results of several cytokines based on the results of MR-Egger intercept (*P_intercept_* > 0.05) and MR-PRESSO global test (*P_global test_* > 0.05). The detailed data are shown in **Table 4**; **Supplementary Table 4**.

**Table 3:** Primary results for MR analysis of sepsis on circulating cytokines (reverse).

	Method	Beta	SE	OR	95% CI	*P* -value
CTACK levels	Inverse variance weighted	0.088	0.123	1.092	0.859-1.390	0.472
beta-nerve growth factor levels	Inverse variance weighted	-0.193	0.124	0.825	0.647-1.051	0.119
Vascular endothelial growth factor levels	Inverse variance weighted	0.026	0.084	1.026	0.871-1.210	0.756
Macrophage Migration Inhibitory Factor levels	Inverse variance weighted	-0.001	0.120	0.999	0.790-1.263	0.991
TRAIL levels	Inverse variance weighted	0.076	0.081	1.079	0.920-1.264	0.351
Tumor necrosis factor beta levels	Inverse variance weighted	0.071	0.253	1.073	0.653-1.762	0.780
Tumor necrosis factor alpha levels	Inverse variance weighted	-0.076	0.128	0.927	0.722-1.190	0.552
Stromal-cell-derived factor 1 alpha levels	Inverse variance weighted	0.032	0.091	1.033	0.864-1.235	0.723
Stem cell growth factor beta levels	Inverse variance weighted	0.197	0.124	1.218	0.955-1.552	0.112
Stem cell factor levels	Inverse variance weighted	0.100	0.076	1.105	0.951-1.283	0.192
Interleukin-16 levels	Inverse variance weighted	-0.063	0.129	0.939	0.729-1.209	0.626
RANTES levels	Inverse variance weighted	-0.031	0.122	0.970	0.763-1.232	0.802
Platelet-derived growth factor BB levels	Inverse variance weighted	0.103	0.076	1.108	0.956-1.285	0.174
Macrophage inflammatory protein 1b levels	Inverse variance weighted	0.049	0.117	1.050	0.835-1.320	0.678
Macrophage inflammatory protein 1a levels	Inverse variance weighted	-0.069	0.124	0.934	0.732-1.190	0.579
Monokine induced by gamma interferon levels	Inverse variance weighted	-0.050	0.176	0.951	0.674-1.343	0.777
Macrophage colony stimulating factor levels	Inverse variance weighted	-0.003	0.216	0.997	0.653-1.522	0.989
Monocyte chemoattractant protein-3 levels	Inverse variance weighted	-0.376	0.459	0.687	0.279-1.688	0.413
Monocyte chemoattractant protein-1 levels	Inverse variance weighted	0.029	0.085	1.029	0.871-1.216	0.737
Interleukin-12p70 levels	Inverse variance weighted	0.006	0.082	1.006	0.856-1.182	0.943
Interferon gamma-induced protein 10 levels	Inverse variance weighted	-0.049	0.126	0.952	0.743-1.220	0.698
Interleukin-18 levels	Inverse variance weighted	0.074	0.113	1.077	0.863-1.344	0.510
Interleukin-17 levels	Inverse variance weighted	0.090	0.085	1.094	0.927-1.292	0.289
Interleukin-13 levels	Inverse variance weighted	-0.239	0.126	0.787	0.615-1.007	0.057
Interleukin-10 levels	Inverse variance weighted	-0.021	0.093	0.979	0.816-1.174	0.817
Interleukin-8 levels	Inverse variance weighted	-0.069	0.114	0.934	0.746-1.168	0.548
Interleukin-6 levels	Inverse variance weighted	-0.045	0.083	0.956	0.813-1.125	0.591
Interleukin-1-receptor antagonist levels	Inverse variance weighted	-0.202	0.124	0.817	0.641-1.042	0.104
Interleukin-1-beta levels	Inverse variance weighted	-0.146	0.094	0.864	0.719-1.038	0.119
Hepatocyte growth factor levels	Inverse variance weighted	-0.072	0.079	0.931	0.798-1.086	0.361
Interleukin-9 levels	Inverse variance weighted	-0.158	0.124	0.854	0.669-1.090	0.205
Interleukin-7 levels	Inverse variance weighted	-0.096	0.154	0.909	0.673-1.228	0.534
Interleukin-5 levels	Inverse variance weighted	-0.164	0.150	0.848	0.632-1.139	0.274
Interleukin-4 levels	Inverse variance weighted	0.059	0.083	1.061	0.902-1.248	0.473
Interleukin-2 receptor antagonist levels	Inverse variance weighted	-0.037	0.117	0.963	0.766-1.212	0.750
Interleukin-2 levels	Inverse variance weighted	-0.007	0.127	0.993	0.775-1.272	0.954
Interferon gamma levels	Inverse variance weighted	0.062	0.088	1.064	0.896-1.263	0.482
Growth-regulated protein alpha levels	Inverse variance weighted	-0.010	0.115	0.990	0.791-1.240	0.931
Granulocyte-colony stimulating factor levels	Inverse variance weighted	0.055	0.083	1.057	0.899-1.243	0.502
Fibroblast growth factor basic levels	Inverse variance weighted	0.046	0.085	1.047	0.886-1.238	0.588
Eotaxin levels	Inverse variance weighted	-0.043	0.086	0.958	0.810-1.133	0.618

MR, mendelian randomization; SE, standard error; OR, odds ratio; CI, confdence interval; CTACK, cutaneous T-cell attracting (CCL27); RANTES, regulated on activation, normal T-cell expressed and secreted (CCL5); TRAIL, TNF-related apoptosis-inducing ligand.

**Table 4:** Heterogenity and pleiotropy analyses of sepsis on circulating cytokines (reverse).

	Heterogenity		MR-Egger intercept		
	Q	Q_pvalue	Egger_intercept	SE	*P* -value
CTACK levels	8.626	0.375	0.027	0.025	0.311
beta-nerve growth factor levels	10.155	0.338	-0.021	0.026	0.451
Vascular endothelial growth factor levels	7.820	0.552	0.017	0.017	0.350
Macrophage Migration Inhibitory Factor levels	3.086	0.929	-0.003	0.025	0.922
TRAIL levels	5.240	0.813	-0.011	0.017	0.538
Tumor necrosis factor beta levels	2.919	0.571	-0.175	0.143	0.309
Tumor necrosis factor alpha levels	6.101	0.636	0.013	0.027	0.641
Stromal-cell-derived factor 1 alpha levels	11.099	0.269	-0.010	0.020	0.620
Stem cell growth factor beta levels	7.917	0.442	0.035	0.026	0.213
Stem cell factor levels	4.236	0.895	-0.012	0.016	0.470
Interleukin-16 levels	5.774	0.673	0.025	0.028	0.395
RANTES levels	9.402	0.401	0.027	0.025	0.312
Platelet-derived growth factor BB levels	9.541	0.482	0.008	0.016	0.639
Macrophage inflammatory protein 1b levels	23.445	0.009	0.010	0.025	0.701
Macrophage inflammatory protein 1a levels	3.523	0.940	0.025	0.026	0.362
Monokine induced by gamma interferon levels	16.230	0.059	0.027	0.038	0.499
Macrophage colony stimulating factor levels	13.500	0.061	0.032	0.048	0.523
Monocyte chemoattractant protein-3 levels	9.322	0.054	-0.095	0.295	0.769
Monocyte chemoattractant protein-1 levels	3.110	0.875	0.015	0.018	0.439
Interleukin-12p70 levels	2.570	0.958	0.007	0.017	0.678
Interferon gamma-induced protein 10 levels	10.775	0.291	-0.008	0.028	0.767
Interleukin-18 levels	5.590	0.848	0.021	0.023	0.392
Interleukin-17 levels	9.080	0.430	0.007	0.019	0.726
Interleukin-13 levels	4.788	0.780	0.037	0.026	0.198
Interleukin-10 levels	9.412	0.309	-0.001	0.021	0.957
Interleukin-8 levels	8.135	0.616	0.030	0.024	0.234
Interleukin-6 levels	7.137	0.522	0.006	0.017	0.745
Interleukin-1-receptor antagonist levels	3.290	0.915	0.000	0.026	0.993
Interleukin-1-beta levels	9.218	0.417	0.041	0.019	0.062
Hepatocyte growth factor levels	4.966	0.761	-0.022	0.016	0.226
Interleukin-9 levels	4.223	0.836	0.027	0.026	0.326
Interleukin-7 levels	9.510	0.218	0.038	0.032	0.275
Interleukin-5 levels	12.607	0.181	0.076	0.027	0.021
Interleukin-4 levels	9.211	0.418	0.008	0.018	0.668
Interleukin-2 receptor antagonist levels	3.139	0.925	0.005	0.024	0.840
Interleukin-2 levels	1.905	0.984	0.000	0.026	0.989
Interferon gamma levels	11.091	0.270	-0.007	0.019	0.737
Growth-regulated protein alpha levels	7.117	0.714	0.038	0.024	0.146
Granulocyte-colony stimulating factor levels	3.133	0.959	-0.006	0.017	0.720
Fibroblast growth factor basic levels	4.690	0.860	0.004	0.018	0.835
Eotaxin levels	2.731	0.909	0.000	0.018	0.996

SE, standard error; CTACK, cutaneous T-cell attracting (CCL27); RANTES, regulated on activation, normal T-cell expressed and secreted (CCL5); TRAIL TNF-related apoptosis-inducing ligand.

## Discussion

In the present study, we utilized the two-sample MR method to investigate the potential causal associations between circulating levels of 41 cytokines and the risk of sepsis. We found suggestive evidence that the genetically predicted circulating levels of RANTES, basic-FGF and β-NGF were associated with sepsis. These findings provide new insights into the pathogenesis of sepsis.

RANTES/CCL5 is a chemokine that plays an important role in inflammation by recruiting leukocytes including monocytes, memory T cells, eosinophils and NK cells to sites of inflammation (32). Little is known about the role of RANTES in sepsis, and the studies that are currently available have inconsistent results regarding the role of RANTES. In basic research, Ness et al. found in a mouse sepsis model that the use of CCL5 increased sepsis-induced lethality in wild-type mice, while neutralization of CCL5 improved survival (33), in contrast to a recent study by Xie et al. who demonstrated that RANTES levels were significantly elevated in septic mice (34). In some small-sample clinical observational studies it was indicated that RANTES concentrations were significantly lower in the plasma of septic neonates and could be used as a biomarker for predicting neonatal infections (35–37). However, Cavaillon et al. found that RANTES levels were significantly higher in sepsis survivors than in non-survivors; moreover, RANTES levels did not correlate with any other cellular levels (including IL-6, IL-8, monocyte chemotactic protein-1 [MCP-1], etc.), and negatively correlated with acute physiologic assessment and chronic health evaluation II (APACHE II) scores, with low levels being significantly predictive of poor outcome (38). These findings do not entirely align with our own research. The specific role of RANTES in sepsis is currently unclear, as various studies have produced conflicting results and there are doubts about its clinical significance. These discrepancies may be due to variations in the animal models used, the timing and dosage of RANTES administration, and other factors that can affect its effects. Additionally, the limited sample size in current clinical studies may have contributed to individual differences. Nevertheless, our MR analysis suggests that circulating RANTES may have a potential protective effect against sepsis, although further studies are needed to validate these findings. Importantly, our inverse MR analysis did not identify a causal relationship between sepsis and basal levels of RANTES. Mechanistically, RANTES plays a crucial role in recruiting immune cells, such as monocytes, to sites of infection in order to eliminate pathogens and provide protection against infections (32). Additionally, it is capable of facilitating T cell proliferation and differentiation (39). Furthermore, elevated levels of RANTES may counteract immunosuppression in sepsis, particularly in advanced stages, by supporting the recovery of T lymphocytes (40). Given these functions, further research is necessary to fully understand the exact mechanisms through which RANTES contributes to the development of sepsis.

Basic-FGF, also known as FGF-2, is a member of the fibroblast growth factor family (41). It is a potent mitogen involved in wound healing, angiogenesis and embryonic development. Basic-FGF stimulates the proliferation, differentiation and migration of many cell types involved in inflammation and tissue repair, including endothelial cells, fibroblasts, smooth muscle cells and keratinocytes (42). In addition to its effects on cell growth and motility, basic-FGF also regulates the production of inflammatory mediators, extracellular matrix components and proteinases (43). Through its diverse biological activities, basic-FGF plays a key role in mediating inflammatory responses, angiogenesis and wound healing (44). Currently, there is only one clinical study that compares blood FGF-2 concentrations in 118 healthy control subjects with 18 sepsis patients. This study found that the median FGF-2 concentration in sepsis patients was significantly lower than that in healthy controls (25.7 *vs.* 37.7, *P* =0.0057) (45). Other studies have focused on basic research. For example, Pan et al. found that FGF-2 had a therapeutic effect on sepsis-associated acute lung injury by improving capillary leakage and reducing inflammatory response through cellular and animal experiments (46). Similarly, Sun et al. suggested that FGF-2 inhibited coagulant activity in septic mice, reduced lung and liver injuries, and improved survival, highlighting the role of FGF-2 in ameliorating sepsis-induced coagulation abnormalities (47). Moreover, in sepsis-induced cardiac injury, delivery of basic-FGF via nanoparticles as a carrier has been shown to treat sepsis-induced cardiac injury and protect cardiomyocytes from oxidative and inflammatory damage (48). Although an observational study found that basic-FGF levels were lower in sepsis patients compared to the normal population, analysis of prognosis was absent. Evidence from experimental studies supports our finding of a potential protective effect of basic-FGF in sepsis. These data suggest that basic-FGF may be a potential therapeutic target for sepsis, but further studies are warranted to confirm the underlying biological mechanisms. Specifically, large-scale clinical studies analyzing the prognostic value of basic-FGF levels in sepsis patients are needed. Additionally, more research is required to elucidate the complex immunomodulatory effects of basic-FGF in sepsis through *in vitro* and animal models. Gaining a better understanding of how basic-FGF regulates inflammatory pathways and mediates organ damage and recovery will be crucial in determining its viability as a sepsis treatment. Overall, basic-FGF shows promise as a therapeutic target in sepsis, but clinical and mechanistic validation is still lacking.

β-NGF, also known as nerve growth factor beta (NGFB), is a vital component of the nerve growth factor family. It is synthesized by multiple types of cells and plays a crucial role in the development, maintenance, and survival of neurons in the central and peripheral nervous systems (49). By binding to tropomyosin receptor kinase A (TrkA) and p75 neurotrophin receptor (p75NTR), β-NGF activates signaling cascades that regulate neuronal differentiation, neurite outgrowth, and nociception (50). In inflammation, β-NGF elicits hypersensitivity to pain and heat by increasing neurotransmitter release from damaged sensory neurons (51). It also stimulates endothelial cells to enhance adhesion molecule expression and facilitate leukocyte infiltration (52). Additionally, β-NGF triggers mast cell degranulation and histamine release, while activating production of inflammatory mediators like substance P and calcitonin gene-related peptide (CGRP) (53). Overall, β-NGF plays a crucial role as a mediator of neuroinflammation and development of inflammatory hyperalgesia, exerting pro-inflammatory effects on both nerve and immune cells. To date, there have been no direct studies examining the relationship between β-NGF and sepsis. However, Boucly et al. discovered that serum β-NGF levels can serve as a predictor of death or lung transplantation in patients with pulmonary arterial hypertension (54). Additionally, a study by Hepburn et al. revealed that β-NGF plays a key regulatory role in the immune response to S. aureus infection by enhancing phagocytosis and superoxide-dependent killing, stimulating proinflammatory cytokine production, and promoting calcium-dependent neutrophil recruitment (55). These findings provide indirect support for our result that genetically predicted higher β-NGF levels increase sepsis risk. This association may be due to the fact that β-NGF is connected to excessive inflammatory response and cellular damage in sepsis. Further research is still needed to directly investigate the mechanistic links between β-NGF and sepsis pathogenesis.

In summary, the role of RANTES in sepsis has produced inconsistent results in previous research. However, our MR analysis suggests that RANTES may have a protective effect, although further validation is necessary. Mechanistically, RANTES recruits immune cells to the infection sites, promotes T cell proliferation, and may counteract immunosuppression in late-stage sepsis. Future research should focus on determining its effects on inflammatory pathways in sepsis and confirming its prognostic value through extensive clinical studies. Moreover, basic-FGF shows promise as a therapeutic target, as experimental studies have demonstrated its protective effects against sepsis-associated organ damage and coagulation abnormalities. However, there is a lack of clinical research, and further work is needed to validate the prognostic value and mechanisms of basic-FGF in sepsis using human samples. Understanding its immunomodulatory functions may provide support for the potential use of basic-FGF as a treatment. Finally, it is suggested that β-NGF may contribute to excessive inflammation and cellular injury in sepsis, although there is currently no direct evidence linking it to sepsis pathogenesis. Moving forward, it is crucial for studies to investigate the direct involvement of β-NGF in sepsis development through clinical evaluations and mechanistic experiments.

This study has numerous strengths. Firstly, it employs MR to establish causal inferences while minimizing confounding bias, giving it a significant advantage over conventional observational studies. Secondly, it explores a wide range of 41 cytokines, offering a comprehensive overview of potential cytokine mediators in sepsis. Thirdly, it uncovers new connections between RANTES, basic-FGF, β-NGF, and sepsis risk, providing valuable insights into novel pathogenic mechanisms. Lastly, it proposes logical directions for future research to validate and build upon these findings.

However, it is important to acknowledge the limitations of this study. Firstly, our selection of IVs utilized a relaxed significance threshold of *P* < 5 × 10^-6^, which introduces the potential for false-positive variants and subsequent bias. However, it is worth noting that the IVs consistently demonstrated F-statistics greater than 10, indicating a weak instrumental bias is less likely. Secondly, the focus on a single ethnicity (European) limits the generalizability of our findings to other populations. Thirdly, while efforts were made to mitigate confounding, it is impossible to completely rule out the presence of pleiotropy. Lastly, we did not examine potential downstream mechanisms that could elucidate the connection between the identified cytokines and the pathogenesis of sepsis.

## Conclusions

This MR study provides first novel evidence that genetically predicted causal association of circulating levels of RANTES, basic FGF, and β-NGF with altered sepsis risk. The findings shed light on the potential involvement of these cytokines in sepsis pathogenesis. Further experimental research is warranted to validate the observed associations and elucidate the underlying biological mechanisms linking RANTES, basic-FGF, β-NGF to sepsis development. Clinical studies with diverse patient populations are also needed to confirm the prognostic utility of these cytokines. Although requiring additional confirmation, the results contribute new insights into cytokine mediators in sepsis and suggest promising future research directions.

## Data availability statement

The original contributions presented in the study are included in the article/**Supplementary Material**. All data used in the current study are publicly available GWAS summary data.

## Author contributions

SL: Conceptualization, Funding acquisition, Methodology, Writing – original draft, Writing – review & editing. XM: Data curation, Software, Visualization, Writing – original draft. WH: Software, Visualization, Writing – original draft.

## Supplementary material

Supplementary Figure 1 Scatter plot of β-NGF levels on sepsis.

Supplementary Figure 2 Leave-one-out plot of β-NGF levels on sepsis.

Supplementary Figure 3 Scatter plot of RANTES levels on sepsis.

Supplementary Figure 4 Leave-one-out plot of RANTES levels on sepsis.

Supplementary Figure 5 Scatter plot of basic-FGF levels on sepsis.

Supplementary Figure 6 Leave-one-out plot of basic-FGF levels on sepsis.
