# Supplementary figures and images for: Causal association of circulating cytokines with sepsis: a Mendelian randomization study
Source: Front Immunol. 2023 Oct 17;14:1281845. doi: 10.3389/fimmu.2023.1281845 (PMC10616607; doi:10.3389/fimmu.2023.1281845)

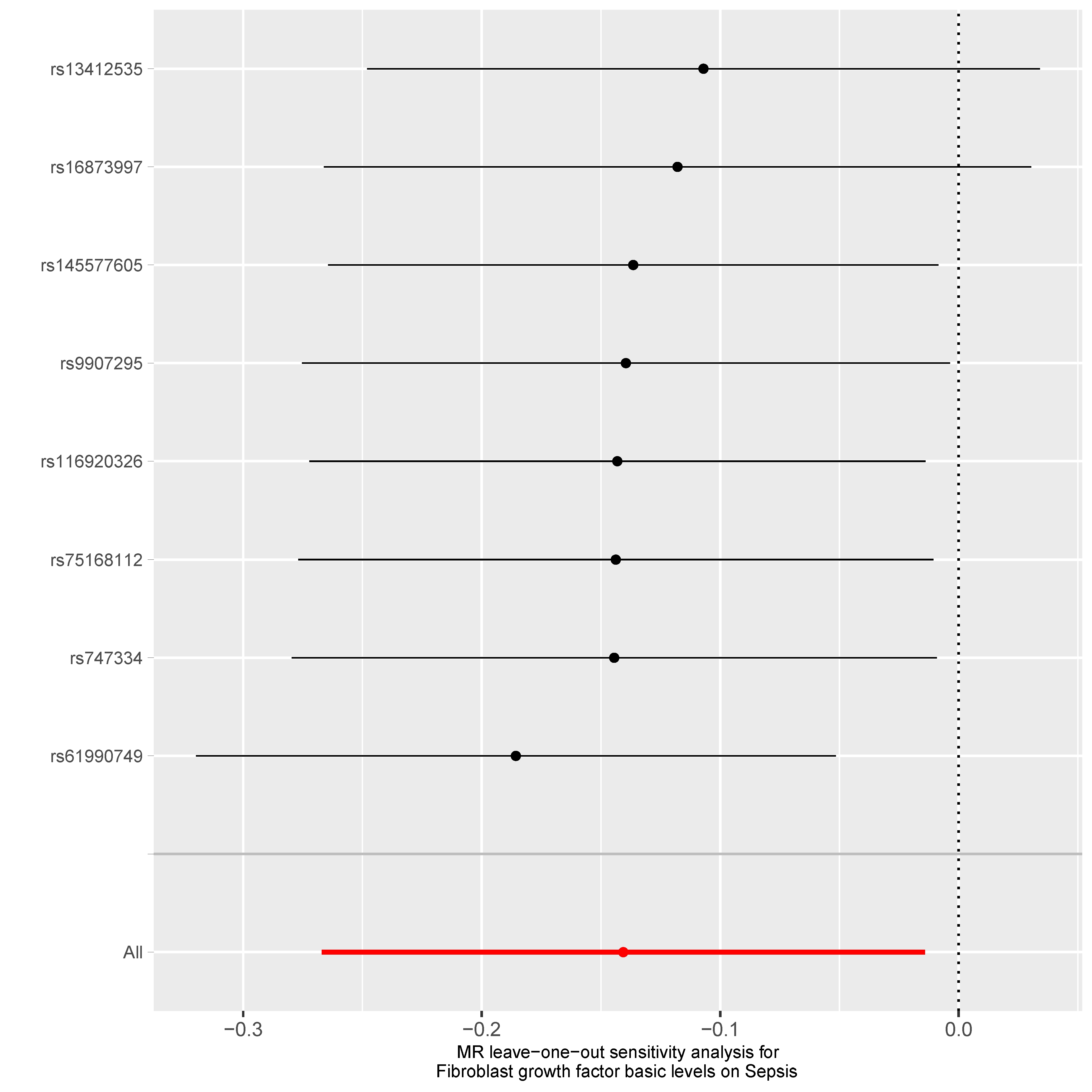

Supplement: Supplementary Figure 1 — Scatter plot of β-NGF levels on sepsis. [file Image_1.tiff]

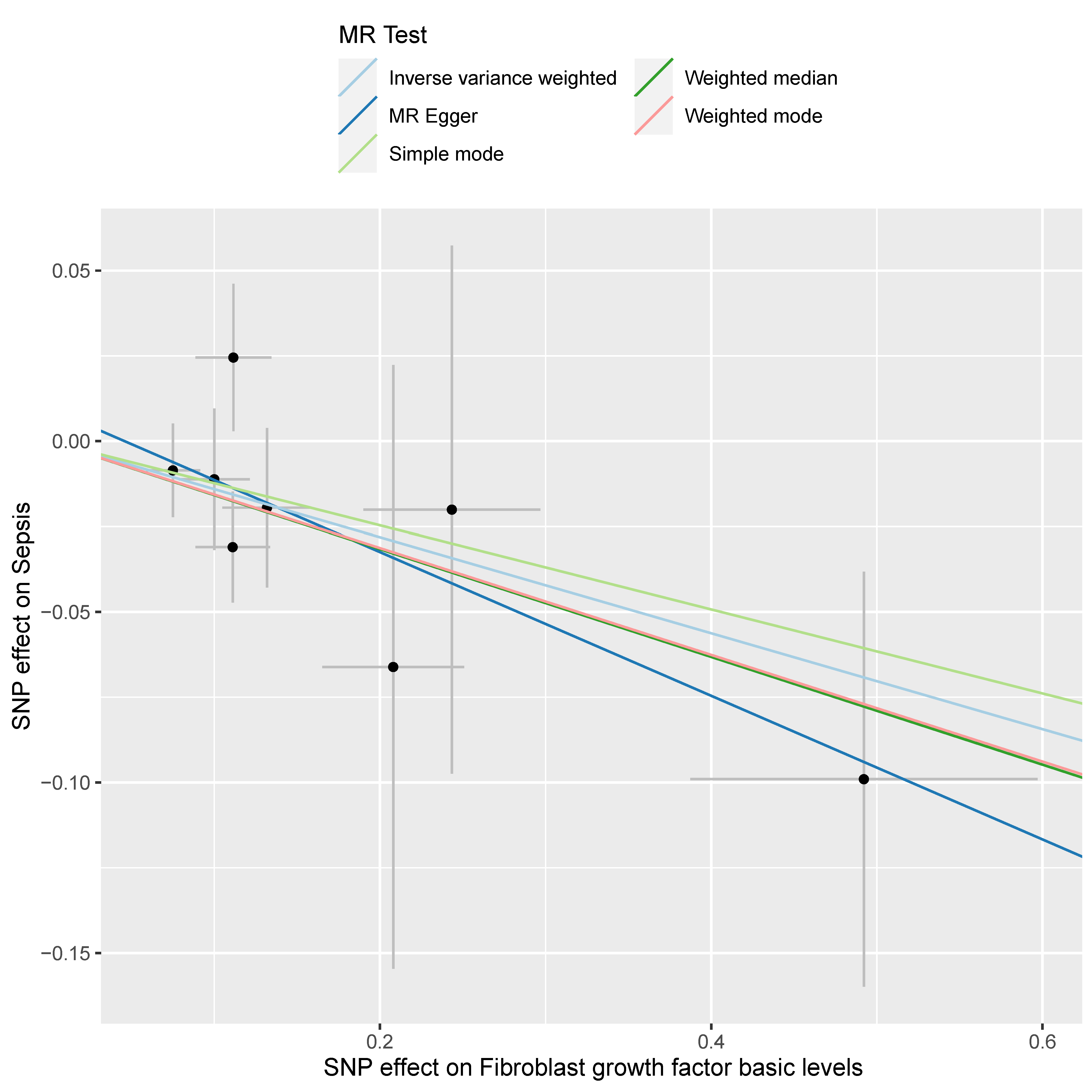

Supplement: Supplementary Figure 2 — Leave-one-out plot of β-NGF levels on sepsis. [file Image_2.tiff]

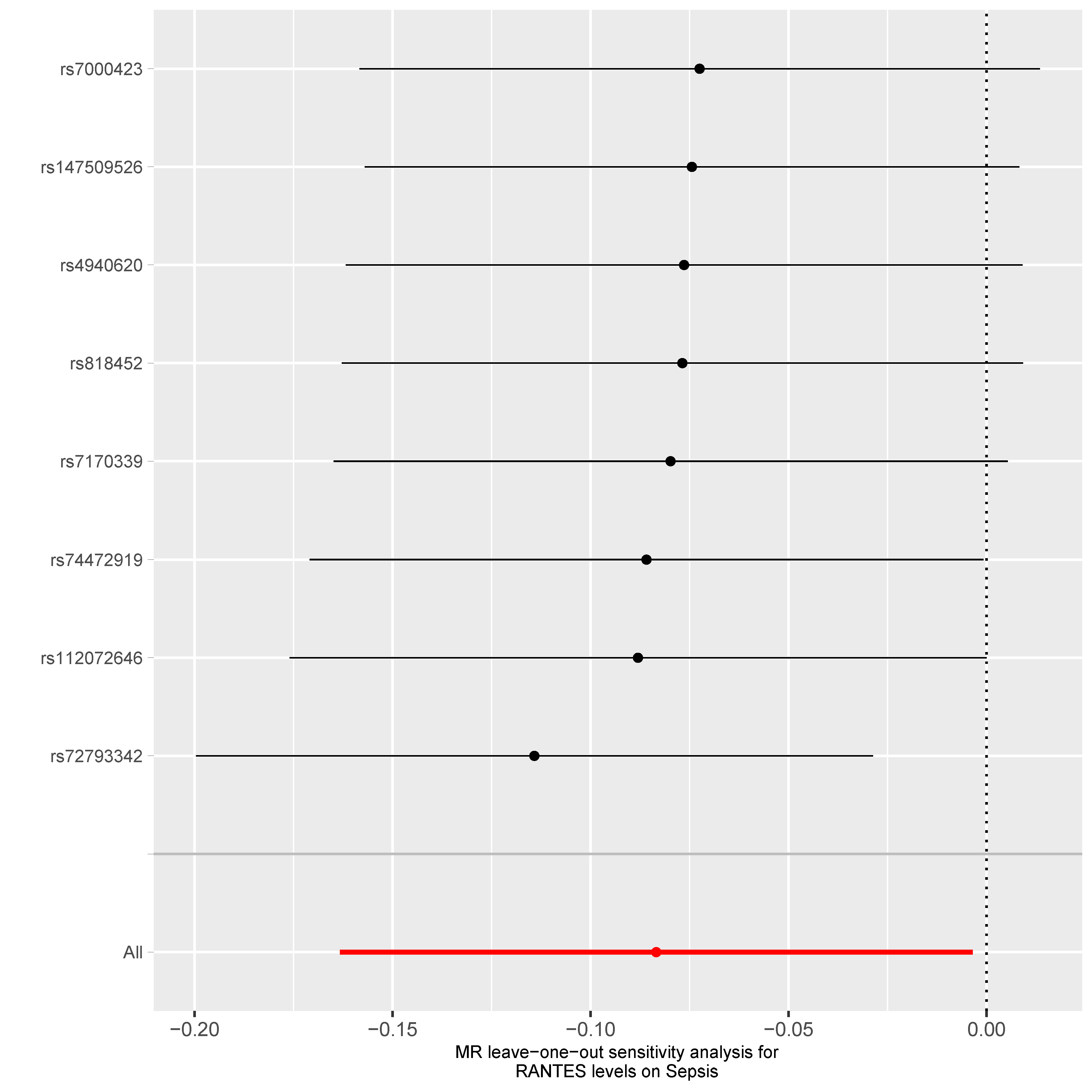

Supplement: Supplementary Figure 3 — Scatter plot of RANTES levels on sepsis. [file Image_3.tiff]

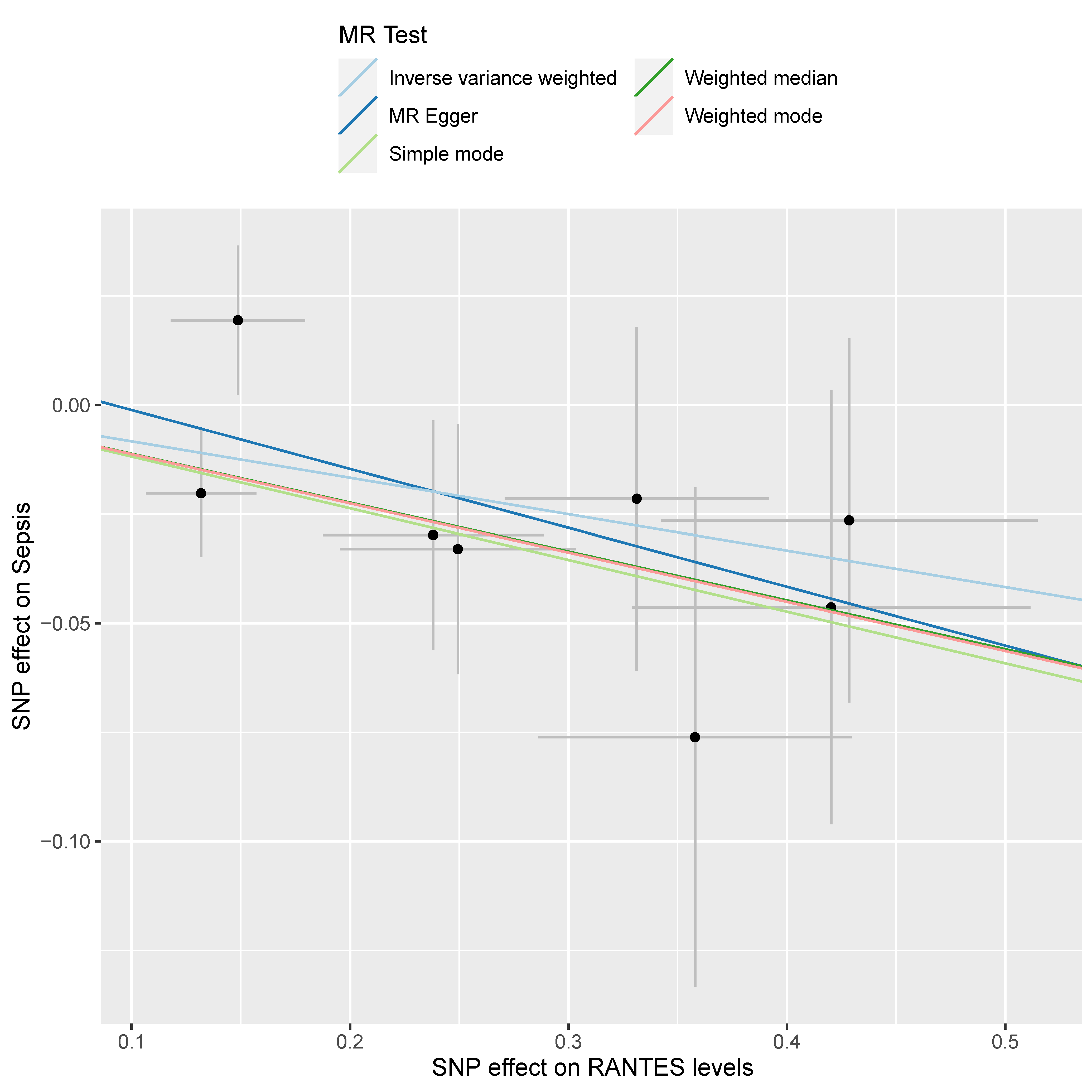

Supplement: Supplementary Figure 4 — Leave-one-out plot of RANTES levels on sepsis. [file Image_4.tiff]

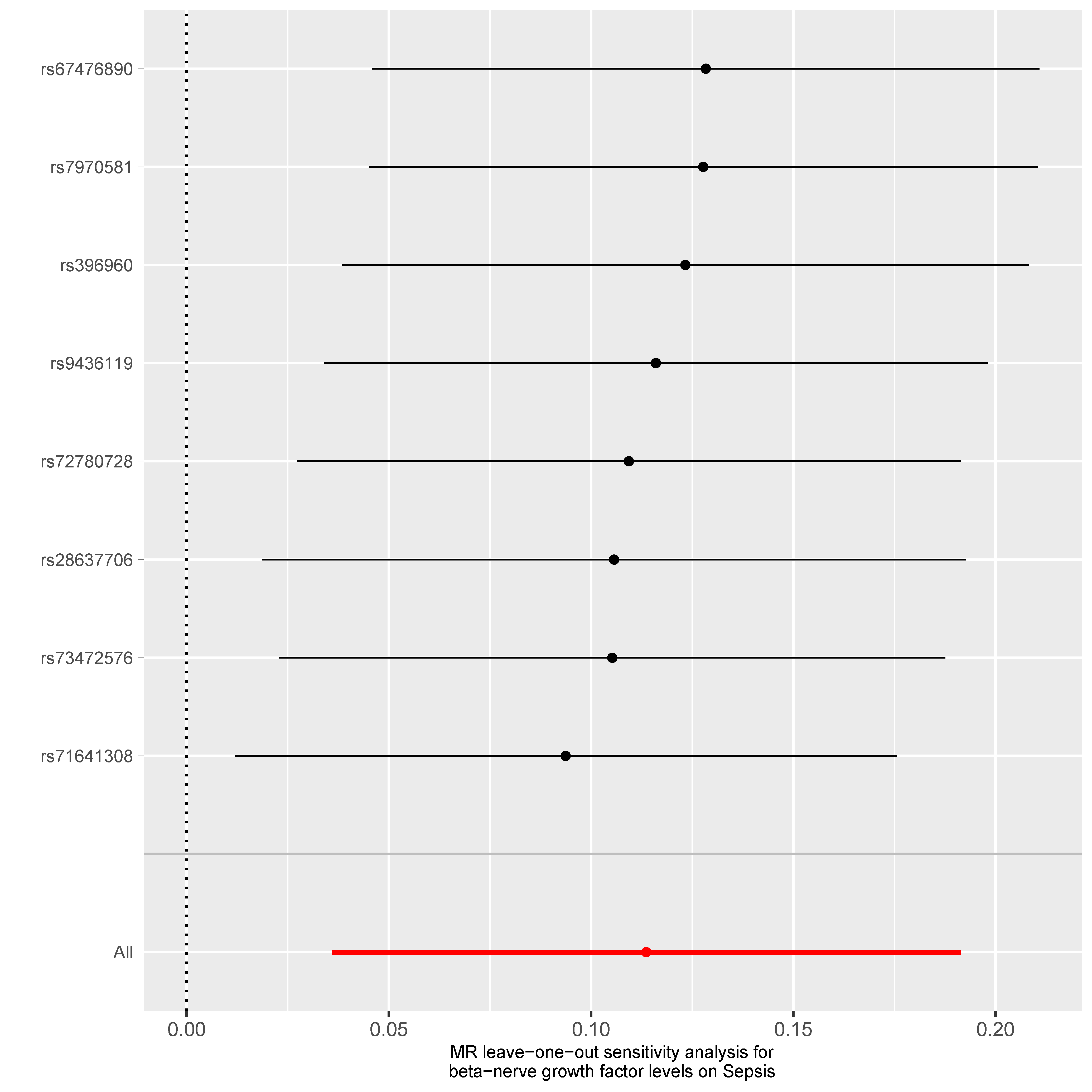

Supplement: Supplementary Figure 5 — Scatter plot of basic-FGF levels on sepsis. [file Image_5.tiff]

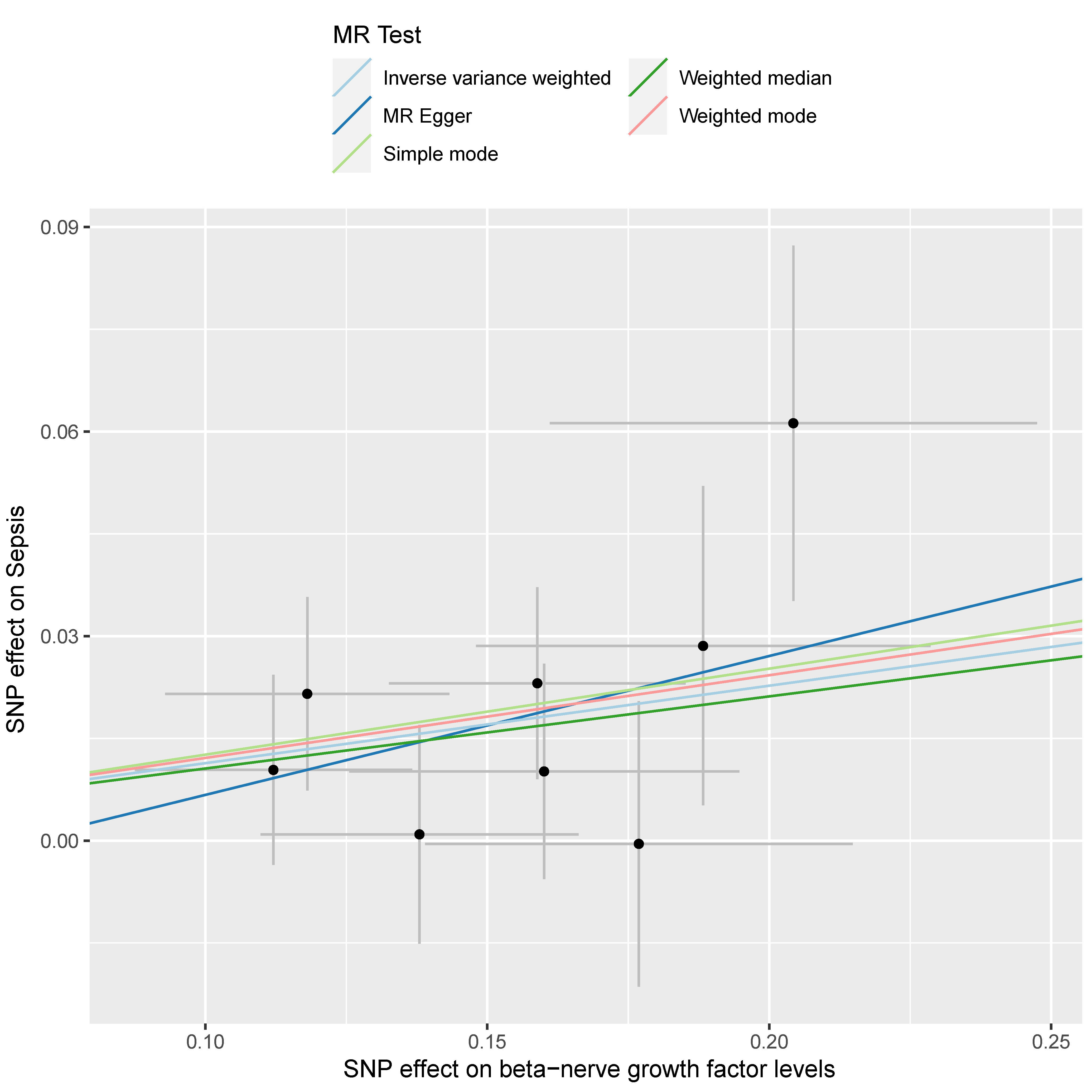

Supplement: Supplementary Figure 6 — Leave-one-out plot of basic-FGF levels on sepsis. [file Image_6.tiff]
